# Analysis and Compensation of Modulation Angular Rate Error Based on Missile-Borne Rotation Semi-Strapdown Inertial Navigation System

**DOI:** 10.3390/s18051430

**Published:** 2018-05-04

**Authors:** Jiayu Zhang, Jie Li, Xi Zhang, Xiaorui Che, Yugang Huang, Kaiqiang Feng

**Affiliations:** 1 Key Laboratory of Instrumentation Science & Dynamic Measurement, Ministry of Education, North University of China, Taiyuan 030051, China; 18734196406@163.com (J.Z.); Zhangxi@nuc.edu.cn (X.Z.); Xiaorui0630@163.com (X.C.); b1506011@st.nuc.edu.cn (K.F.); 2 National Key Laboratory for Electronic Measurement Technology, North University of China, Taiyuan 030051, China; 3 The 60th Research Institute of General Staff Dept of P.L.A, Nanjing 210000, China; hyg34217@163.com

**Keywords:** Rotation Semi-SINS, rotation modulation, rotating angular rate, compensation

## Abstract

The Semi-Strapdown Inertial Navigation System (SSINS) provides a new solution to attitude measurement of a high-speed rotating missile. However, micro-electro-mechanical-systems (MEMS) inertial measurement unit (MIMU) outputs are corrupted by significant sensor errors. In order to improve the navigation precision, a rotation modulation technology method called Rotation Semi-Strapdown Inertial Navigation System (RSSINS) is introduced into SINS. In fact, the stability of the modulation angular rate is difficult to achieve in a high-speed rotation environment. The changing rotary angular rate has an impact on the inertial sensor error self-compensation. In this paper, the influence of modulation angular rate error, including acceleration-deceleration process, and instability of the angular rate on the navigation accuracy of RSSINS is deduced and the error characteristics of the reciprocating rotation scheme are analyzed. A new compensation method is proposed to remove or reduce sensor errors so as to make it possible to maintain high precision autonomous navigation performance by MIMU when there is no external aid. Experiments have been carried out to validate the performance of the method. In addition, the proposed method is applicable for modulation angular rate error compensation under various dynamic conditions.

## 1. Introduction

The independent high-precision measurement of a high-speed rotating missile’s attitude is the key technology of guidance and precision strikes, which is the main development trend of conventional high-speed rotating missile guidance [1]. However, the common Strap-down Inertial Navigation System (SINS) is not suitable for highly dynamic and highly spinning missiles, due to the fact the noise properties of a typical MEMS gyroscope lead to large accumulative errors after integration from angular rates to attitude angles [2]. In recent years, the measurement of missiles’ attitude has attracted a lot of attention. Thus, the concept of the Gyroscope-free Strapdown Inertial Navigation System (GF-SINS) which uses a configuration of accelerometers only to measure the acceleration and rotational motion of a rigid body in 3D space has been proposed and extensively researched. In principle, it benefits from an effect known as lever-arm effect [3]. Parsa et al. obtained the nine-element angular information vector (AIV) using twelve accelerometers. In that work, the complete AIV is used, but the focus is on the optimal design of the gyro-free inertial measurement unit (GF-IMU), avoiding the use of tri-axial accelerometers, and not on the fusion scheme [4]. In [5], Edwan et al. presented an extended Kalman filter (EKF)-based solution for the estimation of the angular motion using a GFIMU built of twelve separate mono-axial accelerometers. Constrained Kalman filtering has proven to be an efficient technique for improving the estimated state vector if the constraints are handled properly. In general, the GF-SINS is a medium and low precision navigation system. An absence of gyroscopes leads to the accelerated growth of errors and high-precision measurements are hard to achieve in short time.

Meanwhile, vector measurements play a central role, without using gyros, in the problem of attitude determination as discussed in a recent survey [6,7,8,9]. Magnis et al. considered a nonlinear observer that directly uses the vector measurements to reconstruct the angular velocity. A nonlinear observer with extended state and output injection is defined and its convergence is proved by identifying the error equation as a linear time-varying (LTV) system perturbed by a linear-quadratic term [10]. However, sensors producing vector measurements, such as Sun sensors, magnetometers and star sensors, are susceptible to the external environment which makes it difficult to employ them in all terrains and at all times.

In addition, [11] proposed a method to estimate the linear velocity of shells. Transverse accelerometers are used to measure the aerodynamic forces motivated by the pitching and yawing motion. Then, the coupled pitching and yawing dynamics are deduced, so that the linear velocity can be estimated through the dependency of the natural frequency of these coupled pendulum-like dynamics. However, the signals from sensors are corrupted not only by noises, but also by parasitic sensed motions caused by their locations inside the shell.

**Figure 1:**
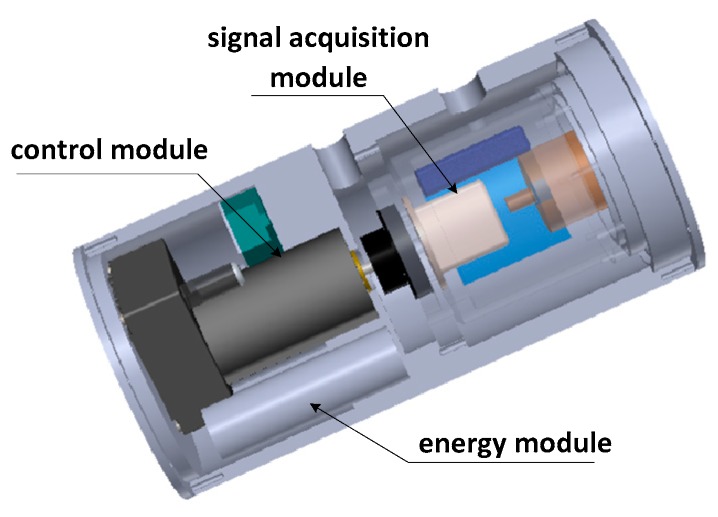
Arrangement of Semi-Strapdown INS.

We still choose the MIMU to measure the velocity, position and attitude information. Different from traditional measurement methods, however, the concept of Semi-Strapdown INS was proposed by the Key Laboratory of Instrumentation Science & Dynamic Measurement [12,13]. Figure 1 shows the structure of a Semi-Strapdown INS system. It is consist of three parts, including energy module, control module and signal acquisition module. In contrast to SINS, the signal acquisition module isn’t rigidly attached to the carrier, but rather is connected to the carrier via the rotating mechanism, which provides a stable low-dynamic environment for SINS with the rotating mechanism and eliminates the interference of the high-speed rotation on the device accuracy, therefore, gyros with small range can be used to measure the carrier’s attitude information in a relatively stable environment.

MIMU outputs, however, are corrupted with significant sensor errors, such as high frequency noise, bias, scale factors and installation errors. As a result, navigation errors will accumulate quickly and deteriorate the navigation solution over a short time period [14,15,16]. In order to address this issue, rotation modulation technology is introduced into SINS, giving what is then called a Rotation Semi-Strap-down Inertial Navigation System (RSSINS). It is an auto-compensation technique to force the MEMS inertial measurement unit (MIMU) to rotate along given axes regularly, thus modulating the constant gyro drifts and accelerometer biases into periodically varying components. These periodical components can be mitigated through integral calculation, so that navigation errors are attenuated prominently without external aiding information [17,18,19].

In practical applications, for realizing the dual-function of rotation reduction and rotation modulation in a high-speed rotation environment, the rotary mechanism is required to follow the high-dynamic changes of the missile in real-time, which inevitably causes problems such as overshooting and instability of the rotation angular rate, thus resulting in residual errors. Therefore, it is extremely important for the system to analyze and compensate the modulation angular rate errors.

Reference [20] studied the influence of acceleration-deceleration process of modulation angular rate on the modulation effect of inertial sensors’ biases and analyzed the error characteristics of IMU rotation schemes both in backward and forward directions. In [21], Wang analyzed the influence of acceleration-deceleration process on inertial sensor errors of INS include biases, scale factor errors, and installation errors mainly, which can be compensated to a certain degree by the 4-position rotation scheme. Reference [22] studied the angle motion of the rotating mechanism in rotation strapdown inertial navigation systems, and a variable motion model was established. Then, the main errors caused by constant drifts and scale factor errors of IMU were derived. The 4-position reciprocating rotation scheme was designed, and the design principle of rotation scheme was analyzed, although the above analysis did not consider the impact of modulation angular velocity instability on navigation accuracy, and these rotation schemes make it harder to achieve precise control of the rotating mechanism in a highly dynamic environment.

In this paper, the impact of modulation angular rate errors, mainly including the acceleration-deceleration process and the overshoot and instability of the modulation angular rate, on navigation accuracy is analyzed, and a novel compensation method is proposed based on angular rate errors in RSSINS. The effectiveness of the compensation method is proved by an actual RSSINS manufactured by our lab.

The remainder of this paper is organized as follows: Section 2 briefly illustrates the way that system works and establishes the solution model of carrier attitude and position based on the MIMU output information. In Section 3, the impact of modulation angular rate errors on navigation accuracy was analyzed and discussed. Then, the correctness of the analysis is verified by simulation. The compensation method is proposed and the tests are given to verify errors suppression performance of the compensation algorithm in Section 4. Section 5 presents the conclusions.

## 2. Rotary Semi-Strap-Down Inertial Navigation System

### 2.1. The Principle behind the RSSINS

**Figure 2:**
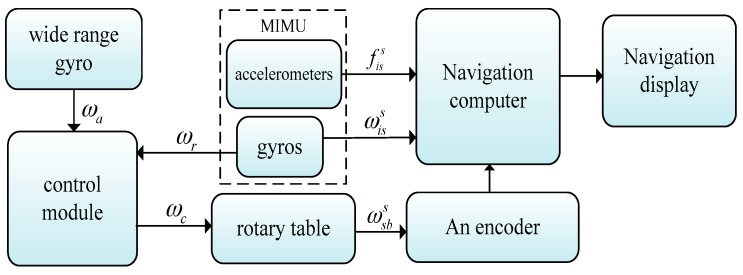
Block diagram of the principle behind the RSSINS.

Distinct from the SSINS, the idea of closed-loop feedback control is proposed in order to achieve the dual function of rotary mechanism, both rotation reduction and rotation modulation of the MIMU in the RSSINS. The principle behind the RSSINS is shown in Figure 2.

One wide range gyro is mounted on the roll-axis of the carrier, which can measure the carrier’s roll-axis angular rate ($\omega_{a}$) in a high-spinning environment. Then, $\omega_{a}$ is fed back to the control module, so as to control the motor to drive the MIMU to rotate in the opposite direction. Due to the low precision of the wide range gyroscope the other small range gyro is installed on signal acquisition module for measuring the residual angular rate ($\omega_{r}$), which is fed back to the control module for calculating the corrected angular rate ($\omega_{c}$) in real time to drive the MIMU to complete the rotation modulation, so the modulation angular rate consists of the residual angular rate and the corrected angular rate.

**Figure 3:**
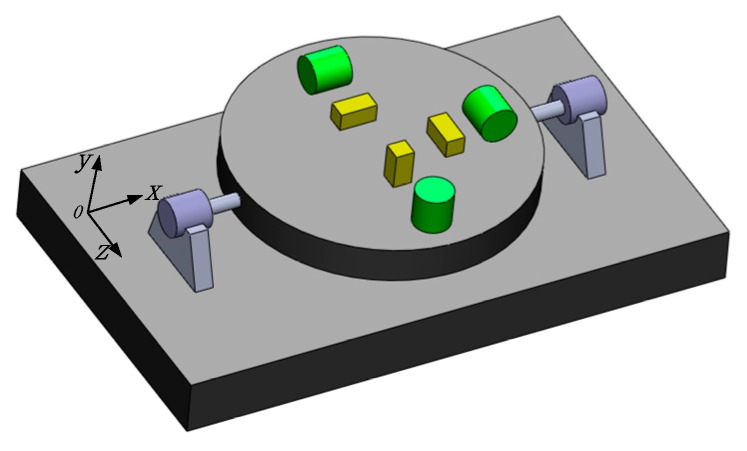
Concept of Isolation of the roll axis.

In this case, isolation of the micro-inertial measurement component and the high-rotation of carrier’s roll-axis is implemented by these mechanical structure and control method. The conceptual schematic of “rotary axial isolation and radial strapdown” is shown as Figure 3.

The rectangle represents the carrier, the cylinders and rectangles set on the disk are the inertial components. The disk is connected to the missile body by the rolling axis. Consequently, the signal acquisition module will not follow carrier to rotate at high speed.

In order to describe the orientation, the coordinate systems are defined as follows: the navigation coordinate system (N-frame) is chosen as the local geographical coordinate frame; the body coordinate system (B-frame) is the carrier coordinate system, in which the X-axis is aligned with the roll-axis, the Y-axis points to the top of the body and the Z-axis refers to the right direction as shown in Figure 3. As the MIMU is rotating in the RSSINS, a new frame in which the inertial readings are collected is introduced. The new coordinate system can be referred as inertial sensor frame (S-frame), and its axis are aligned with the sensitive axis of inertial sensors. At the beginning time, the S-frame is coincident with the B-frame. For the convenience of description, in addition, S’-frame is defined as a virtual coordinate system, which refers to S-frame before introduced the rotation modulation.

**Figure 4:**
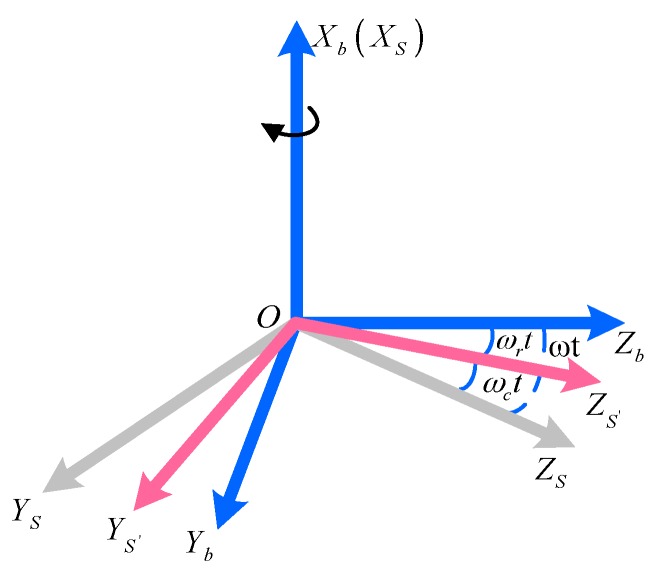
The relationship between B-frame, S’-frame and S-frame.

After reducing the rotation angular rate, the roll-axis angular rate of MIMU is $\omega_{r}$, as shown in Figure 4, and there is a transition matrix from S’-frame to B-frame, denoted by $C_{s^{\prime }}^{b}$. Then the rotation mechanism drives the MIMU with the angular rate of $\omega_{c}$, and there is a transition matrix from S-frame to S’-frame, denoted by $C_{s}^{s^{\prime }}$. To simplify the analysis, we just take one situation into account where the B-frame is aligned with N-frame. Take the gyro as an example, the constant gyro drifts projected on the navigation coordinate frame are expressed as Equation (1) during the rotating process:

(1) $$\left[\begin{array}{c} \varepsilon_{E}\\ \varepsilon_{N}\\ \varepsilon_{U} \end{array}\right]=C_{b}^{n}C_{s^{\prime }}^{b}C_{s}^{s^{\prime }}\varepsilon^{s}=\left[\begin{array}{ccc} 1 & 0 & 0\\ 0 & \cos\omega_{r}t & -\sin\omega_{r}t\\ 0 & \sin\omega_{r}t & \cos\omega_{r}t \end{array}\right]\left[\begin{array}{ccc} 1 & 0 & 0\\ 0 & \cos\omega_{c}t & -\sin\omega_{c}t\\ 0 & \sin\omega_{c}t & \cos\omega_{c}t \end{array}\right]\left[\begin{array}{c} \varepsilon_{x}^{s}\\ \varepsilon_{y}^{s}\\ \varepsilon_{z}^{s} \end{array}\right]=\left[\begin{array}{ccc} 1 & 0 & 0\\ 0 & \cos\omega_{m}t & -\sin\omega_{m}t\\ 0 & \sin\omega_{m}t & \cos\omega_{m}t \end{array}\right]\left[\begin{array}{c} \varepsilon_{x}^{s}\\ \varepsilon_{y}^{s}\\ \varepsilon_{z}^{s} \end{array}\right]$$

According to Equation (1), the integration of gyro drifts in navigation frame for a complete rotation cycle can be described by Equation (2):

(2) $$\int \varepsilon^{n}=\left[\begin{array}{c} T\varepsilon_{x}^{s}\\ 0\\ 0 \end{array}\right]$$

From the above analysis we can see, the single-axis rotation modulation is a technique where by rotating MIMU around an axis periodically according to some rule makes the constant drifts and some other constant or slowly varying errors of inertial sensor perpendicular to the rotation axis be modulated into periodic components. Therefore, their integration for a complete cycle is zero or some value close to zero and they will not accumulate in the navigation solution [23,24] and the error on the rotation axis is propagated according to the original law.

### 2.2. Solution Algorithm

**Figure 5:**
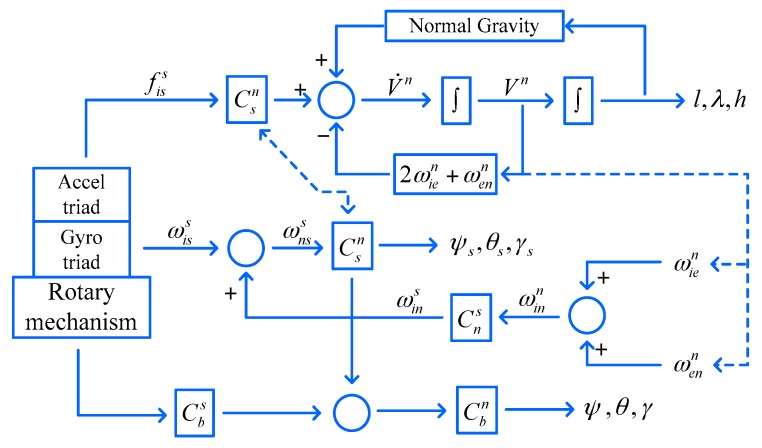
Flowchart of rotary semi-strapdown solution algorithm.

Generally speaking, the mechanization algorithm of RSSINS is similar to the conventional INS one. Nevertheless, the inertial sensor outputs are collected from S-frame in RSSINS. In the integrated design of “semi-strapdown” and “rotary modulation”, the solution algorithm is designed based on the relationship between MIMU output information and carrier motion parameters. The principle of rotary semi-strapdown solution algorithm is shown in Figure 5.

In this solution algorithm, the attitude matrix update is carried out between the MIMU coordinate system and the geographic coordinate system. The angular rate and specific force measured by the MIMU are directly introduced into the solution algorithm without the corresponding transformation. In this case, the attitude matrix is $C_{s}^{n}$, as shown in Figure 5, from which the MIMU attitude (denoted by $\psi_{s},\theta_{s},\gamma_{s}$) can be obtained. The carrier attitude needs to be calculated according to the angular position of the rotary mechanism. $C_{b}^{s}$ is constructed though relative rotation angle measured by an encoder, and the transition matrix from B-frame to N-frame $C_{b}^{n}$ can be got, from which the MIMU attitude (denoted by ψ,θ,γ) can be calculated. In addition, the measurement error of the relative rotation angle will result in the attitude error of carrier roll-axis in the solution structure, but it will not be introduced into the solution loop and have no effect on the velocity and position accuracy [25].

## 3. Analysis of Modulation Angular Rate Errors

Rotation modulation is essentially an error self-compensation technique by periodic rotation of an inertial sensitive unit, effectively improving the navigation accuracy when the device accuracy is low. While the rotary mechanism is required to rapidly respond in real-time to changes of the carrier, problems such as overshoot and instability of the angular rate are inevitable, so that sensor errors cannot be completely suppressed and the performance of navigation accuracy is decreased.

**Figure 6:**
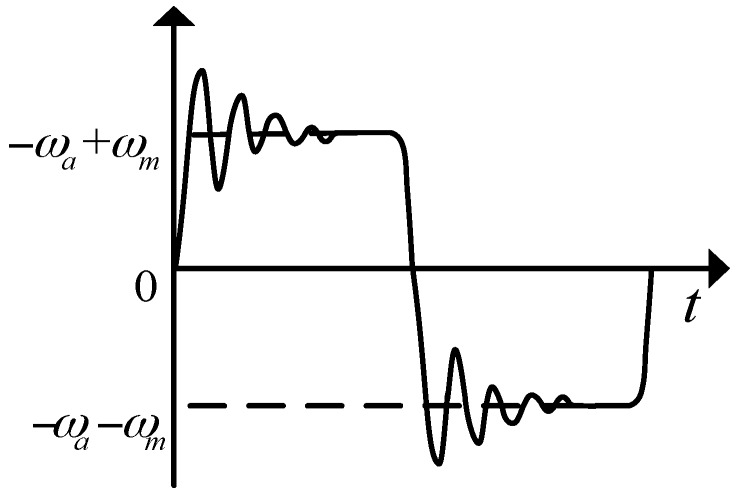
The rotation angular rate of rotary mechanism.

The actual situation of rotary mechanism angular rate is shown as Figure 6, where $\omega_{a}$ is the carrier’s roll-axis angular rate, $\omega_{m}$ is modulation angular rate. The rotation angular rate errors mainly include the acceleration-deceleration process at the beginning and ending of rotating stage and the instability of rotation angular rate in the rotation process. The navigation error caused by them should not be neglected.

The unidirectional continuous rotation motivates significant navigation error, if the scale factor in rotation axis cannot be completely removed [26]. To tackle this issue, the reciprocating rotation scheme is employed. A complete reciprocating rotation cycle includes a rotation cycle (360 deg) about the Z axis in the counter-clockwise (positive) direction and then a rotation cycle about Z axis in the clockwise (negative) direction. In the inertial navigation system, the errors of the gyros are the critical factors determining the accuracy of the system [27], and the error modulation of accelerometer is similar to gyroscope, so taking the gyroscope as an example, the influence of rotational angular rate error is analyzed in detail.

### 3.1. Acceleration-Deceleration Process

**Figure 7:**
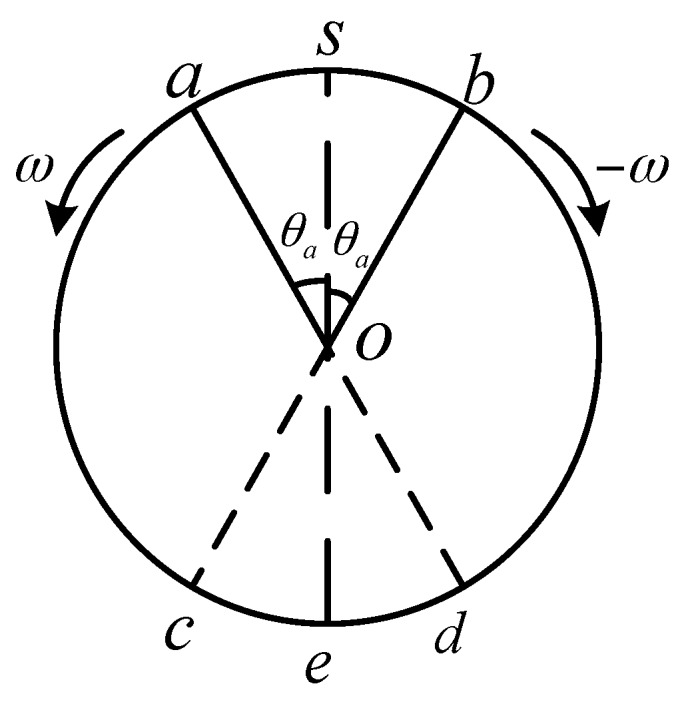
The rotation process in a reciprocating rotation cycle.

First, regardless of the instability of the rotational angular rate, the effects of acceleration-deceleration process of rotation angular rate on constant bias, scale factor and installation error are analyzed in a reciprocating rotation cycle. The rotation process is shown in Figure 7.

The positive rotation process is described as follows. Starting from S, when accelerating to position a, MIMU reaches the modulation angular rate, and the time taken by it is $t_{a}$, the turned angle at which the acceleration process is $\theta_{a}$. Then the motor drives the MIMU to rotate at a constant angular rate of $\omega_{m}$ for a period of $t_{c}$ reached position *b*. Starting from position b, MIMU has a deceleration process, and the time taken by it is $t_{a}$, the angle at which the acceleration process is $\theta_{a}$. Similar to the positive rotation process, the negative rotation process takes the same time. So, in a reciprocating rotation process, a complete modulation cycle is: $T=4t_{a}+2t_{c}$, where $\theta_{a}=\frac{1}{2}\alpha \cdot t_{a}^{2}$, $t_{c}=\frac{\left(2\pi -2\theta_{a}\right)}{\omega }$.

#### 3.1.1. Constant Drifts

During the acceleration process, the gyro drifts can be modulated as Equation (3). And the attitude errors caused by gyro drifts are expressed as Equation (4):

(3) $$\delta \omega_{sa}^{n}=C_{b}^{n}C_{s}^{b}\varepsilon^{s}=\left[\begin{array}{ccc} 1 & 0 & 0\\ 0 & \cos\theta_{a} & -\sin\theta_{a}\\ 0 & \sin\theta_{a} & \cos\theta_{a} \end{array}\right]\left[\begin{array}{c} \varepsilon_{x}^{s}\\ \varepsilon_{y}^{s}\\ \varepsilon_{z}^{s} \end{array}\right]=\left[\begin{array}{c} \varepsilon_{x}^{s}\\ \varepsilon_{y}^{s}\cos\theta_{a}-\varepsilon_{z}^{s}\sin\theta_{a}\\ \varepsilon_{y}^{s}\sin\theta_{a}+\varepsilon_{z}^{s}\cos\theta_{a} \end{array}\right]$$

(4) $$\delta \psi_{Dsa}=\int_{0}^{t_{a}}\delta \omega_{sa}^{n}dt=\left[\begin{array}{c} \varepsilon_{x}^{s}t_{a}\\ \varepsilon_{y}^{s}\frac{\sqrt{\pi }FresnelC(\sqrt{\alpha }t_{a}/\sqrt{\pi })}{\sqrt{\alpha }}-\varepsilon_{z}^{s}\frac{\sqrt{\pi }FresnelS(\sqrt{\alpha }t_{a}/\sqrt{\pi })}{\sqrt{\alpha }}\\ \varepsilon_{y}^{s}\frac{\sqrt{\pi }FresnelS(\sqrt{\alpha }t_{a}/\sqrt{\pi })}{\sqrt{\alpha }}+\varepsilon_{z}^{s}\frac{\sqrt{\pi }FresnelC(\sqrt{\alpha }t_{a}/\sqrt{\pi })}{\sqrt{\alpha }} \end{array}\right]$$

where, FresnelC and FresnelS are the so-called Fresnel integrals, which can be approximated as follow, when ξ<1 [21]:

(5) $$\begin{array}{l} FresnelC(\xi )\approx \xi \\ FresnelS(\xi )\approx \frac{\pi }{6}\xi^{3} \end{array}$$

In general, the condition of $\sqrt{\alpha }t_{a}/\sqrt{\pi }<1$ can be satisfied, so according to Equation (4), the attitude errors, during the acceleration phase, can be expressed as follows:

(6) $$\delta \psi_{Dsa}=\left[\begin{array}{c} \varepsilon_{x}^{s}t_{a}\\ \varepsilon_{y}^{s}t_{a}-\varepsilon_{z}^{s}\frac{\alpha t_{a}^{3}}{6}\\ \varepsilon_{z}^{s}t_{a}+\varepsilon_{y}^{s}\frac{\alpha t_{a}^{3}}{6} \end{array}\right]$$

In uniform motion, the gyro drifts can be modulated as Equation (7) and the attitude errors caused by gyro drifts can be expressed as Equation (8). Similarly, the attitude errors, during the deceleration stage, can be expressed as Equation (9):

(7) $$\delta \omega_{ab}^{n}=C_{b}^{n}C_{s}^{b}\varepsilon^{s}=\left[\begin{array}{ccc} 1 & 0 & 0\\ 0 & \cos\left(\theta_{a}+\omega t\right) & -\sin\left(\theta_{a}+\omega t\right)\\ 0 & \sin\left(\theta_{a}+\omega t\right) & \cos\left(\theta_{a}+\omega t\right) \end{array}\right]\left[\begin{array}{c} \varepsilon_{x}^{s}\\ \varepsilon_{y}^{s}\\ \varepsilon_{z}^{s} \end{array}\right]=\left[\begin{array}{c} \varepsilon_{x}^{s}\\ \varepsilon_{y}^{s}\cos\left(\theta_{a}+\omega t\right)-\varepsilon_{z}^{s}\sin\left(\theta_{a}+\omega t\right)\\ \varepsilon_{y}^{s}\sin\left(\theta_{a}+\omega t\right)+\varepsilon_{z}^{s}\cos\left(\theta_{a}+\omega t\right) \end{array}\right]$$

(8) $$\delta \psi_{Dab}=\int_{t_{a}}^{t_{a}+t_{c}}C_{b}^{n}C_{s}^{b}\varepsilon^{s}=\left[\begin{array}{c} \varepsilon_{x}^{s}t_{c}\\ \varepsilon_{y}^{s}\frac{\sin\left(\theta_{a}+\omega t_{c}\right)-\sin\theta_{a}}{\omega }+\varepsilon_{z}^{s}\frac{\cos\left(\theta_{a}+\omega t_{c}\right)-\cos\theta_{a}}{\omega }\\ -\varepsilon_{y}^{s}\frac{\cos\left(\theta_{a}+\omega t_{c}\right)-\cos\theta_{a}}{\omega }+\varepsilon_{z}^{s}\frac{\sin\left(\theta_{a}+\omega t_{c}\right)-\sin\theta_{a}}{\omega } \end{array}\right]$$

(9) $$\delta \psi_{Dbs}=\left[\begin{array}{c} \varepsilon_{x}^{s}t_{a}\\ \varepsilon_{y}^{s}t_{a}-\varepsilon_{z}^{s}\frac{\alpha t_{a}^{3}}{6}\\ \varepsilon_{z}^{s}t_{a}+\varepsilon_{y}^{s}\frac{\alpha t_{a}^{3}}{6} \end{array}\right]$$

In the same way, during the negative rotation the attitude errors caused by gyro drifts can be expressed as Equations (10)–(12):

(10) $$\delta \psi_{Dsb}=\left[\begin{array}{c} \varepsilon_{x}^{s}t_{a}\\ \varepsilon_{y}^{s}t_{a}+\varepsilon_{z}^{s}\frac{\alpha t_{a}^{3}}{6}\\ \varepsilon_{z}^{s}t_{a}-\varepsilon_{y}^{s}\frac{\alpha t_{a}^{3}}{6} \end{array}\right]$$

(11) $$\delta \psi_{Dba}=\left[\begin{array}{c} \varepsilon_{x}^{s}t_{c}\\ \varepsilon_{y}^{s}\frac{\sin(-\theta_{a}-\omega t_{c})+\sin\theta_{a}}{-\omega }+\varepsilon_{z}^{s}\frac{\cos(-\theta_{a}-\omega t_{c})-\cos\theta_{a}}{-\omega }\\ -\varepsilon_{y}^{s}\frac{\cos(-\theta_{a}-\omega t_{c})-\cos\theta_{a}}{-\omega }+\varepsilon_{z}^{s}\frac{\sin(-\theta_{a}-\omega t_{c})+\sin\theta_{a}}{-\omega } \end{array}\right]$$

(12) $$\delta \psi_{Das}=\left[\begin{array}{c} \varepsilon_{x}^{s}t_{a}+\varepsilon_{y}^{s}\frac{\alpha t_{a}^{3}}{6}\\ \varepsilon_{y}^{s}t_{a}-\varepsilon_{x}^{s}\frac{\alpha t_{a}^{3}}{6}\\ \varepsilon_{z}^{s}t_{a} \end{array}\right]$$

It is known from the above analysis that the attitude errors caused by the gyro drifts in a complete cycle (one positive cycle plus one negative cycle) can be described by Equation (13):

(13) $$\delta \psi_{D}=\left[\begin{array}{c} T\varepsilon_{z}^{s}\\ \frac{2\theta_{a}-\sin\theta_{a}}{\pi }T\varepsilon_{y}^{s}\\ \frac{2\theta_{a}-\sin\theta_{a}}{\pi }T\varepsilon_{z}^{s} \end{array}\right]$$

Compared with the ideal modulation process, the acceleration-deceleration process has no influence on the device error in the direction of the rotation axis, which still propagates according to the original law. The gyro drifts in the direction of perpendicular to the rotation axis is effectively suppressed but not completely eliminated. The attitude errors are dependent on the MIMU turned angle ($\theta_{a}$) in the variable angular rate process. From the Equation (13), it can be seen that the attitude errors grow with the increase of $\theta_{a}$, and accumulate over time.

#### 3.1.2. Scale Factors

Although the scale factors are calibrated before use, some residual error always exists in the inertial sensors. In addition, the scale factors vary with time and temperature [28]. Especially in high-overload environments, the overload of the missile during launch can reach 5000 g for RSSINS. It is assumed that the scale factor error is 0.0001, which is equivalent to a 0.5 g error, and the influence on navigation accuracy cannot be ignored. Therefore, the errors caused by scale factors must be considered in the rotation modulation process.

When MIMU rotates with respect to X axis, the gyros sensed the earth rotation rate and the MIMU rotation rate, therefore the theoretical gyro outputs in S-frame is shown as Equation (14), and the attitude errors caused by scale factors can be described by Equation (15):

(14) $$\left[\begin{array}{c} \omega_{isx}^{s}\\ \omega_{isy}^{s}\\ \omega_{isz}^{s} \end{array}\right]=\left[\begin{array}{c} \omega \\ \omega_{ie}\cos L\cos\omega t-\omega_{ie}\sin L\sin\omega t\\ \omega_{ie}\cos L\sin\omega t+\omega_{ie}\sin L\cos\omega t \end{array}\right]$$

(15) $$\delta \omega_{Kg}^{s}=\left[\begin{array}{ccc} K_{gx} & 0 & 0\\ 0 & K_{gy} & 0\\ 0 & 0 & K_{gz} \end{array}\right]\omega_{is}^{s}=\left[\begin{array}{c} K_{gx}\omega \\ K_{gy}\left(\omega_{ie}\cos L\cos\omega t-\omega_{ie}\sin L\sin\omega t\right)\\ K_{gz}\left(\omega_{ie}\cos L\sin\omega t+\omega_{ie}\sin L\cos\omega t\right) \end{array}\right]$$

where $\omega_{ie}$ is the Earth’s rotation rate, $\omega_{is}^{s}$ is the ideal gyros outputs in S-frame, *L* is the latitude, $\delta \omega_{is}^{s}$ is the sensor errors caused by the scale factors, and $K_{gx}$, $K_{gy}$, $K_{gz}$ are the scale factors for gyros along X, Y and Z axes, respectively.

Similar to the analysis method of constant drifts, the attitude errors caused by the scale factors in a complete cycle (one positive cycle plus one negative cycle) can be described by Equation (16):

(16) $$\delta \psi_{K_{g}}=\int_{0}^{T}C_{b}^{n}C_{s}^{b}\delta \omega_{K_{g}}^{s}=\left[\begin{array}{c} 0\\ \omega_{ie}\cos L\left(\frac{1}{2}\left(K_{gy}-K_{gz}\right)+\frac{1}{4\pi }\left(K_{gy}+K_{gz}\right)\left(4\theta_{a}-\sin2\theta_{a}\right)\right)T\\ \omega_{ie}\sin L\left(-\frac{1}{2}\left(K_{gy}-K_{gz}\right)+\frac{1}{4\pi }\left(K_{gy}+K_{gz}\right)\left(4\theta_{a}-\sin2\theta_{a}\right)\right)T \end{array}\right]$$

Compared with the ideal modulation process, the acceleration-deceleration process has no influence on the eastward attitude, but attitude errors in the north and up directions accumulate over time. In addition to coupling with the northward and upward components of the Earth’s angular velocity, the gyro scale factors of $K_{gy}$, $K_{gz}$ are coupled with the turned angle ($\theta_{a}$) in acceleration-deceleration process. It can be seen that the additional errors are dependent on the MIMU turned angle in the acceleration-deceleration process.

#### 3.1.3. Installation Errors

Due to the imperfection of inertial sensor assembling and installation, the three sensitive axes of the sensor triad are not perfectly orthogonal with each other, which cause the sensed inertial value of one axis to project into the two other axes. The errors caused by installation errors will affect the precision of attitude and position, which can be described by Equation (17) in the condition of ideal modulation angular rate:

(17) $$\delta \omega_{E_{g}}^{s}\;=\left[\begin{array}{c} E_{gxz}\omega_{ie}\left(\sin L\cos\omega t+\cos L\sin\omega t\right)+E_{gxy}\omega_{ie}\left(\cos L\cos\omega t-\sin L\sin\omega t\right)\\ E_{gyx}\omega +E_{gyz}\left(\omega_{ie}\cos L\sin\omega t+\omega_{ie}\sin L\cos\omega t\right)\\ E_{gzx}\omega +E_{gzy}\left(\omega_{ie}\cos L\cos\omega t-\omega_{ie}\sin L\sin\omega t\right) \end{array}\right]$$

where $\delta \omega_{E_{g}}^{s}$ is the gyro errors caused by the installation errors, $E_{gij}$ represent the installation error parameter between *i* axis and *j* axis (*i*, *j* = X, Y, Z). The attitude errors for a complete cycle can be described by:

(18) $$\delta \psi_{E_{g}}=\int_{0}^{T}C_{b}^{n}C_{s}^{b}\delta \omega_{E_{g}}^{s}=\left[\begin{array}{c} \omega_{ie}\frac{\left(E_{gxy}\cos L+E_{gxz}\sin L\right)\left(2\theta_{a}-\sin\theta_{a}\right)}{\pi }T\\ \omega_{ie}\sin L\left(\frac{E_{gyz}+E_{gzy}}{2}+\frac{\left(E_{gyz}-E_{gzy}\right)\left(2\theta_{a}-\sin\theta_{a}\right)}{2\pi }\right)T\\ \omega_{ie}\cos L\left(\frac{E_{gyz}+E_{gzy}}{2}-\frac{\left(E_{gyz}-E_{gzy}\right)\left(2\theta_{a}-\sin\theta_{a}\right)}{2\pi }\right)T \end{array}\right]$$

It can be seen from Equation (18) that the acceleration-deceleration process has an impact on the attitude precision in all three directions. $E_{gxy}$, $E_{gxz}$ are respectively coupled with the northward and upward components of the Earth’s rotation rate, and the turned angle ($\theta_{a}$) is coupled with them. In the east and up direction, $E_{gzy}$, $E_{gyz}$ are respectively coupled with the upward and northward components of the Earth’s angular velocity. In addition, there is the coupling of the residual of $E_{gzy}$, $E_{gyz}$ caused by the turned angle in acceleration-deceleration process and Earth’s angular velocity, which reduce the navigation precision of the system.

#### 3.1.4. Simulation

In fact, the decrease of the performance of rotation modulation is due to the asymmetry of the rotation process caused by the MIMU turned angle ($\theta_{a}$) of the acceleration-deceleration process and the instability of modulation angular rate.

In order to verify that position error depends on the MIMU turned angle rather than the angular acceleration and modulation angular rate, the simulation is designed and carried out with the following conditions: (1) the MIMU turned angle is 30°, the modulation angular rate is 60°/s; (2) the MIMU turned angle is 15°, the modulation angular rate is 60°/s; (3) the MIMU turned angle is 30°, the modulation angular rate is 120°/s; The other simulation parameters are shown in Table 1.

**Figure 8:**
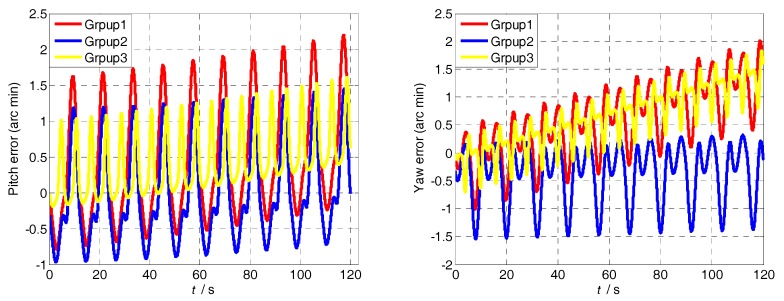
The positioning errors simulation curves of the three groups.

**Figure 9:**
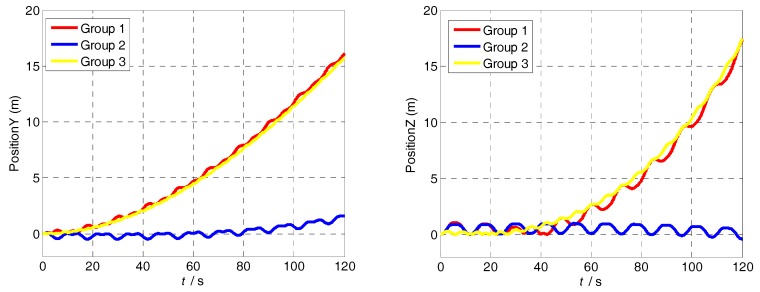
The positioning errors simulation curves of the three groups.

The attitude and position errors simulation curves of the three groups are shown in Figure 8 and Figure 9. It is obvious that the attitude and position error of Group 1 is similar to that of Group 3, and it is larger than Group 2. The difference between Group 2 and other groups is the turned angle in the acceleration-deceleration process. Therefore, the attitude and position accuracy depends on the MIMU turned angle in the acceleration-deceleration process. In addition, compared with Group 1 and Group 3, it is clear that the larger the modulation angular rate, the smaller the error fluctuation.

### 3.2. Instability of Modulation Angular Rate

Due to the RSSINS is applied to the high-rotation environment, the rotation mechanism is required to follow the change of the missile’s rotation speed in real time. In order to satisfy the special application conditions, it is inevitable to generate the fluctuation and overshoot of rotation angular rate, as shown in Figure 4, which degrades the position accuracy.

The attitude errors caused by the instability of rotation angular rate in a complete cycle (one positive cycle plus one negative cycle) can be described by Equation (19):

(19) $$\psi_{D}=\int_{0}^{\frac{T}{2}}C_{s+}^{b}\varepsilon^{s}dt+\int_{\frac{T}{2}}^{T}C_{s-}^{b}\varepsilon^{s}dt$$

where $C_{s+}^{b}$ and $C_{s-}^{b}$ are the direction cosine matrix between the S-frame and the B-frame in the positive and negative rotation process, respectively, and can be described as:

(20) $$C_{s+}^{b}=\left[\begin{array}{ccc} 1 & 0 & 0\\ 0 & \cos\left(\omega +\delta \omega \right)t & -\sin\left(\omega +\delta \omega \right)t\\ 0 & \sin\left(\omega +\delta \omega \right)t & \cos\left(\omega +\delta \omega \right)t \end{array}\right],\;C_{s-}^{b}=\left[\begin{array}{ccc} 1 & 0 & 0\\ 0 & \cos\left(\omega +\delta \omega \right)t & \sin\left(\omega +\delta \omega \right)t\\ 0 & -\sin\left(\omega +\delta \omega \right)t & \cos\left(\omega +\delta \omega \right)t \end{array}\right]$$

where δω is the rotation angular rate error.

According to Equations (19) and (20), the attitude errors caused by the instability of rotation angular velocity error can be expressed by:

(21) $$\delta \psi_{D}=\left[\begin{array}{c} T\cdot \varepsilon_{x}\\ \frac{2\varepsilon_{y}\cdot \sin\left(\frac{2\pi \cdot \delta \omega }{\omega }\right)}{\omega +\delta \omega }\\ \frac{2\varepsilon_{z}\cdot \sin\left(\frac{2\pi \cdot \delta \omega }{\omega }\right)}{\omega +\delta \omega } \end{array}\right]$$

Based on the above analysis, the gyro drift of perpendicular to the rotation axis cannot be completely offset within a positive and negative cycle, and negative cycle and generate the attitude errors in north and up direction. In a reciprocating rotation period, divide attitude errors by the period, the equivalent drifts of rotation modulation can be achieved:

(22) $$\frac{1}{T}\delta \psi_{N}=\xi \cdot \varepsilon_{y},\;\frac{1}{T}\delta \psi_{N}=\xi \cdot \varepsilon_{y}$$

The expression of ξ is:

(23) $$\xi =\frac{\sin(2\pi /K)}{2\pi (1+1/K)}$$

**Figure 10:**
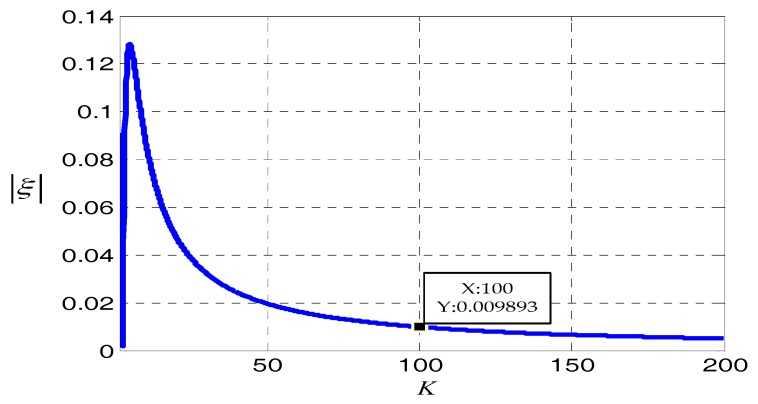
The variation of modulation angular rate error coefficient.

ξ can be named as the modulation angular rate error coefficient of the system, and supposing $K=\frac{\omega }{\delta \omega }$, is the rate between the ideal modulation angular rate of MIMU and the angular rate error of the rotation mechanism. Since the signs of $\varepsilon_{y}$ and $\varepsilon_{z}$ are unknown, the absolute values of ξ can be used to measure the performance of rotation modulation. The curve of rotation modulation angular rate error coefficient will be as in Figure 10.

It can be determined from the changes of the curve of the absolute values of ξ that when the larger K is, the smaller the influence on the modulation effect is. When *K* > 100, the decreasing trend of the absolute values of ξ is gradually slow and ξ is less than 0.01, which indicates that the modulation angular rate error has little impact on the effect of rotation modulation. When *K* continues to increase, it requires higher control accuracy, which is more difficult to achieve. Furthermore, it contributes little to improve the modulation effect. Therefore, when the angular rate error is controlled at one percent of the ideal modulation angular rate, its influence on rotation modulation can be neglected.

## 4. Method and Experimental Results

The essence of rotation modulation technology is that the MIMU has the same movement process at the symmetrical position to offset the sensor errors. As shown in Figure 7, there are acceleration-deceleration processes and the over-shoot of angular rate in a-b phase, and in the c-d phase, which is symmetrical with a-b phase, the MIMU moves in uniform motion. It is obvious that the rotation speed error influence the symmetry of MIMU movement process, which causes the constant biases of inertial sensors cannot be completely eliminate.

The attitude errors caused by the residual sensor drifts can be compensated by the multi-position scheme. However, it is not suitable for high-spinning environment. So, a compensation method of rotation speed error is proposed to achieve error suppression and optimize navigation algorithm in this section.

### 4.1. Compensate Method

The modulation angular rate error makes MIMU unable to reach ideal position at the corresponding moment, and the gyro drifts cannot be modulated into the ideal periodic quantity, thus causing the attitude error. For the convenience of analysis, we define the ideal sensors coordinate system is S_0_-frame.

Due to the modulation angular rate error, $\varepsilon^{s}$ is modulated into the form as shown in Equation (24):

(24) $${\tilde{C}}_{s}^{n}\varepsilon^{s}=C_{s_{0}}^{n}C_{s}^{s_{0}}\cdot \varepsilon^{s}=\left[\begin{array}{ccc} 1 & 0 & 0\\ 0 & \cos\omega t & -\sin\omega t\\ 0 & \sin\omega t & \cos\omega t \end{array}\right]\cdot \left[\begin{array}{ccc} 1 & 0 & 0\\ 0 & \cos\delta \omega t & -\sin\delta \omega t\\ 0 & \sin\delta \omega t & \cos\delta \omega t \end{array}\right]\left[\begin{array}{c} \varepsilon_{x}^{s}\\ \varepsilon_{y}^{s}\\ \varepsilon_{z}^{s} \end{array}\right]\;$$

where $C_{s}^{s_{0}}$ is a transition matrix from S-frame to the ideal sensors coordinate system (S_0_-frame). Obviously, $C_{s}^{s_{0}}$ makes it impossible for the gyro drifts to be modulated into the ideal periodic quantity and the attitude errors caused by residual sensor drifts accumulate over time.

Therefore, the compensation method based on rotation speed error is proposed. The rotation speed error is obtained by the rotation mechanism for building the rotation speed error matrix $C_{s}^{s_{0}}$. Then, the outputs of sensors are compensated by $C_{s}^{s_{0}}$, so as to make the MIMU reach the ideal position through algorithm compensation in the rotation process, and complete the periodic modulation of gyro drifts. Meanwhile the sensor output of rotation-axis is also compensated accordingly. At this point, the attitude matrix constructed by the compensated angular rate is $C_{s_{0}}^{n}$.

The attitude and position information of the carrier are resolved by the solution algorithm mentioned in Section 2.2. During the solution algorithm, the update of attitude matrix is carried out between S_0_-frame and N-frame. Furthermore, $C_{b}^{s}$ is constructed though relative rotation angle measured by an encoder. In this way, the carrier attitude matrix after the algorithm compensation is:

(25) $${\hat{C}}_{b}^{n}=C_{s_{0}}^{n}C_{b}^{s}=C_{b}^{n}C_{s_{0}}^{b}C_{s_{0}}^{s}C_{b}^{s_{0}}=C_{b}^{n}C_{s_{0}}^{b}C_{b}^{s_{0}}C_{s_{0}}^{s}=C_{b}^{n}C_{s_{0}}^{s}$$

Through calculation and analysis, matrix $C_{s_{0}}^{s}$ and $C_{b}^{s_{0}}$ satisfy the commutative law of multiplication. However, the compensation method based on rotation speed error brings some error on the carrier attitude as shown in Equation (25). The error caused by compensation method is analyzed as follows.

Suppose the attitudes of the carrier are denoted as: the pitch angle is θ, the roll angle is γ, and the yaw angle is ψ. The real attitude matrix from the B-frame to N-frame can be expressed by Equation (26):

(26) $$C_{b}^{n}=\left[\begin{array}{ccc} \cos\theta \cos\psi & \cos\theta \sin\psi & -\sin\theta \\ -\cos\gamma \sin\psi +\sin\gamma \sin\theta \cos\psi & \cos\gamma \cos\psi +\sin\gamma \sin\theta \sin\psi & \sin\gamma \cos\theta \\ \sin\gamma \sin\psi +\cos\gamma \sin\theta \cos\psi & -\cos\psi \sin\gamma +\sin\psi \sin\theta \cos\gamma & \cos\gamma \cos\theta \end{array}\right]$$

According to Equation (25), the ${\hat{C}}_{b}^{n}$ can be derived as:

(27) $${\hat{C}}_{b}^{n}=C_{b}^{n}C_{s_{0}}^{s}=\left[\begin{array}{ccc} \cos\theta \cos\psi & \cos\theta \sin\psi & -\sin\theta \\ -\cos\left(\gamma +\delta \omega \cdot t\right)\sin\psi +\sin\left(\gamma +\delta \omega \cdot t\right)\sin\theta \cos\psi & \cos\left(\gamma +\delta \omega \cdot t\right)\cos\psi +\sin\left(\gamma +\delta \omega \cdot t\right)\sin\theta \sin\psi & \sin\left(\gamma +\delta \omega \cdot t\right)\cos\theta \\ \sin\left(\gamma +\delta \omega \cdot t\right)\sin\psi +\cos\left(\gamma +\delta \omega \cdot t\right)\sin\theta \cos\psi & -\cos\psi \sin\left(\gamma +\delta \omega \cdot t\right)+\sin\psi \sin\theta \cos\left(\gamma +\delta \omega \cdot t\right) & \cos\left(\gamma +\delta \omega \cdot t\right)\cos\theta \end{array}\right]$$

It can be seen from Equation (27) that the yaw angle and pitch angle of the carrier are only related to the first row elements of the transformation matrix and the compensation method has no influence on them. However, it has impact on the measurement of roll angle. The roll angle can be deduced from Equation (28). And the measurement error of roll angle caused by compensation method can be calculated as Equation (29):

(28) $$\hat{\gamma }=\arctan\left(\frac{\sin\left(\gamma +\delta \omega \cdot t\right)\cos\theta }{\cos\left(\gamma +\delta \omega \cdot t\right)\cos\theta }\right)=\gamma +\delta \omega \cdot t$$

(29) $$\delta \gamma =\hat{\gamma }-\gamma =\delta \omega \cdot t$$

Finally, the roll angle error can be compensated based on the rotation speed error.

### 4.2. Tests and Results Analysis

In order to further prove the practicability and effectiveness of the proposed method in actual application, experiments are implemented by using a high-precision tri-axial flight simulator. The tri-axial flight simulator has three rotational frames, namely, outer frame, middle frame and inner frame. Table 2 and Table 3 summarize the technical parameters of tri-axial flight simulator and the characteristics of the MIMU in the system, respectively.

**Figure 11:**
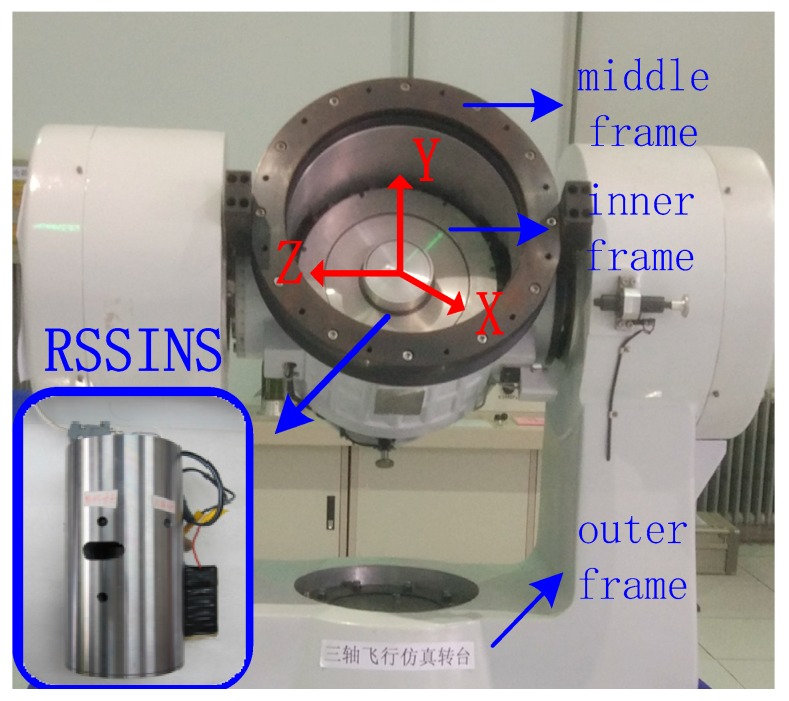
RSSINS installation on tri-axial rotation table.

The impact of the modulation angular rate error to navigation accuracy will be tested and the test results will be compared with the one implemented with a turntable. The system is installed on the turntable, as shown in Figure 11. With the IMU sensitive axes defined as the X axis pointing forward, the Z axis pointing right, and the Y axis pointing up, the rotation of the inner frame rotates the system about its X axis. The experimental conditions are set as follows:

Experiment 1: The inner frame is rotated at high speed to simulate the flight environment for the system so as to realize the dual function of rotation reduction and rotation modulation by rotary mechanism. The roll angular rate of inner frame is set to 7200 deg/s. The Y-axis and the Z-axis respectively measure the pitch and yaw motion of the carrier simulated by the turntable.

Experiment 2: The inner frame directly provides the modulation angular rate for RSSINS, rather than the rotary mechanism, in order to demonstrate the performance of error suppression when the modulation angular rate is steady. Similarly, the Y-axis and the Z-axis respectively measure the pitch and yaw motion of the carrier simulated by the turntable.

Experiment 3: Using the proposed method to compensate the data of Experiment 1, prove the availability of this method.

The experiment conditions are set as shown in Table 4. The pitch and yaw motion of the carrier are driven by high-precision turntable in all the experiments as same.

**Figure 12:**
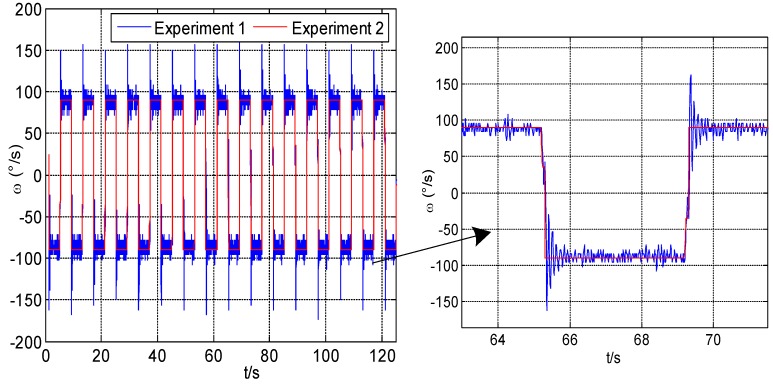
Modulation angular rate of Experiment 1 and Experiment 2.

Figure 12 shows the modulation angular rate of Experiment 1 and Experiment 2, respectively. It can be seen that the angular rate provided by the rotary mechanism has poor stability and overshoot when modulation angular rate changes.

**Figure 13:**
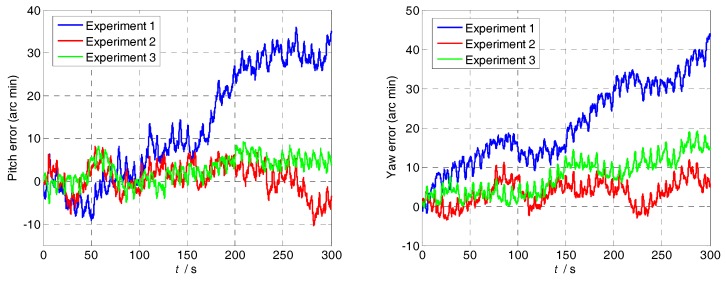
Pitch and yaw error curves of the three experiments.

**Figure 14:**
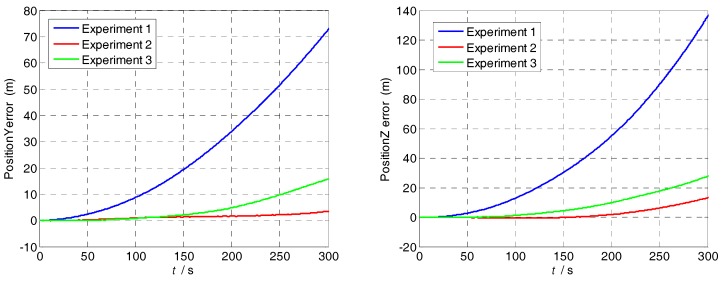
Position errors curves of the three experiments.

Though the analysis of experimental results, the attitude and position errors are shown in Figure 13 and Figure 14. The changes of the attitude angle error before and after compensation are as follows:

The pitch angle error reduces from 34.52 to 4.522′; and the yaw angle error reduces from 43.85 to 14.73′. The position and attitude accuracy increased by more than 65% by using the method in Section 4.1. The proposed method can compensate the modulation angular rate error effectively and it is suitable for various dynamic conditions.

Based on the results from the experiments, we can see that MIMU rotations can modulate the sensor errors and mitigate their effect on the navigation solutions. In addition, the modulation angular rate error makes constant drift unable to be completely offset and the navigation error caused by it accumulates over time. Therefore, their compensation is necessary for a MEMS-based rotary system in order to achieve better navigation performance.

## 5. Conclusions

This paper investigates the impact of modulation angular rate errors on the rotation modulation effect and navigation accuracy. The errors caused by acceleration-deceleration processes and the instability of modulation angular rate are analyzed, and the related mathematical equations are derived and analyzed for a reciprocating rotation scheme. A compensation method is proposed to reduce the effect of modulation angular rate error based on the rotation RSSINS. The experiment results show that the position and attitude accuracy increased by more than 65%. It provides a new method for the attitude and position measurement of the rotary missile. However, the proposed method depends on the output of the rotation mechanism’s speed in real-time. The next step is to study the relationship between the rotating mechanism’s angular rate and the sensitive amount of the MEMS device so as to propose the compensation method of modulation angular rate errors without relying on the output of the rotating mechanism.

## Figures and Tables

**Table 1:** The performance of inertial measurement element.

Error Terms	Parameters
$\varepsilon_{x}=\varepsilon_{y}=\varepsilon_{z}=\varepsilon$	25°/h
$\nabla_{x}=\nabla_{y}=\nabla_{z}=\nabla$	1 mg
$K_{gx}=K_{gy}=K_{gz}$	10^−5^
$\delta KG_{ij}$	0.000025 rad
$\delta KA_{ij}$	0.000015 rad

**Table 2:** Technical parameters of tri-axial flight simulator.

Position Accuracy (°)	Rotation Rate Accuracy (°/s)	Rotation Rate (°/s)		
		Inner Frame	Middle Frame	Outer Frame
0.001	0.001	0.001~12,000	0.001~400	0.001~400

**Table 3:** Characteristics of MIMU.

Characteristics	Range	Bias	Random Walk
Gyroscope (X axis)	±200°/s	24°/h	0.28${}^{\circ }/\sqrt{h}$
Gyroscopes (Y, Z axis)	±75°/s	24°/h	0.28${}^{\circ }/\sqrt{h}$
Accelerometer (X axis)	±200 g	5 mg	150 ug/$\sqrt{Hz}$
Accelerometer (Y, Z axis)	±10 g	1 mg	90 ug/

**Table 4:** Setting of the experiment conditions.

	Pitch	Yaw	MIMU Roll Angular Rate	Rotating Mechanism
Experiment 1	+30 deg–−30 deg	0 deg	90 deg/s	the RSSINS
Experiment 2	+30 deg–−30 deg	0 deg	90 deg/s	High-precision turntable
Experiment 3	+30 deg–−30 deg	0 deg	90 deg/s	Algorithm compensation
